# Mechanisms and Efficacy of Contrast Therapy for Musculoskeletal Painful Disease: A Scoping Review

**DOI:** 10.3390/jcm14051441

**Published:** 2025-02-21

**Authors:** Giulia Leonardi, Simona Portaro, Demetrio Milardi, Francesco Bonanno, Ilaria Sanzarello, Daniele Bruschetta, Cristiano Sconza, Adriana Tisano, Jacopo Maria Fontana, Angelo Alito

**Affiliations:** 1 Physical Rehabilitation Medicine Department, University Hospital A.O.U. “G. Martino”, 98124 Messina, Italy; giulia.leonardi@polime.it (G.L.); simonaportaro@hotmail.it (S.P.); 2 Department of Biomedical, Dental Sciences and Morphological and Functional Images, University of Messina, 98125 Messina, Italy; dmilardi@unime.it (D.M.); ilaria.sanzarello@unime.it (I.S.); dbruschetta@unime.it (D.B.); alitoa@unime.it (A.A.); 3 Department of Clinical and Experimental Medicine, University of Messina, 98125 Messina, Italy; ciccio.bonanno@yahoo.it (F.B.); atisano@unime.it (A.T.); 4 Department of Biomedical Sciences, Humanitas University, 20072 Milan, Italy; cristiano.sconza@humanitas.it; 5 IRCCS Humanitas Research Hospital, 20089 Milan, Italy; 6 Research Laboratory in Biomechanics and Rehabilitation, San Giuseppe Hospital, IRCCS, Istituto Auxologico Italiano, 28921 Verbania, Italy

**Keywords:** contrast bath, contrast therapy, cryo-thermal, heat and cold therapy, musculoskeletal injury, thermal shock

## Abstract

**Background**: Contrast therapy (CT) is a non-pharmacological treatment that alternates between cryotherapy and thermotherapy. It helps reduce VAS pain, improve joint ROM, enhance function, alleviate muscle soreness, and manage swelling, while also improving blood circulation. This scoping review summarizes recent studies on its use for musculoskeletal injuries (e.g., exercise-induced muscle damage, ankle sprain), degenerative conditions (e.g., osteoarthritis), and painful disorders (e.g., complex regional pain syndrome), assessing its healing potential compared to other conservative therapies. **Methods**: PubMed, Scopus, and Cochrane Library were searched to identify relevant publications. Articles were selected using the following inclusion criteria: randomized controlled trials, written in English, published between 2004 and 2024, and addressing the use of CT in the management of musculoskeletal painful conditions. **Results**: Data from 7 articles and 303 patients with musculoskeletal painful conditions treated with CT were included. There was considerable heterogeneity in terms of treatment protocols, with significant differences in the application method, duration, sequence of individuals in each hot/cold cycle, total treatment time, and the pathologies studied. Nevertheless, all studies showed an improvement in the patients’ initial clinical conditions. **Conclusions**: This review highlights the lack of guidelines for the clinical use of CT in musculoskeletal painful conditions. The heterogeneity of the studies reviewed (different clinical scores, follow-up periods, data, and samples) makes the results imprecise. In addition, the modest quality of the trials does not allow the authors to draw clear conclusions about the effectiveness of CT compared with other therapies.

## 1. Introduction

The International Association for the Study of Pain (IASP) [1] defines pain as a “direct or potential damage or an uncomfortable feeling and emotional experience”, often associated with physical, physiological, and psychological disability.

This definition highlights the multidimensional nature of pain, which goes beyond nociception to include cognitive and affective components. In musculoskeletal conditions, pain has a variety of etiologies, including degenerative conditions such as osteoarthritis, which involves progressive cartilage degeneration and joint inflammation, resulting in persistent pain and functional impairment [2]. Acute injuries, such as ligament sprains or muscle strains, trigger inflammatory and nociceptive responses that contribute to localized pain and disability [3]. In addition, chronic musculoskeletal pain syndromes, such as complex regional pain syndrome or fibromyalgia, often involve central sensitization mechanisms, resulting in increased pain perception and altered pain modulation [4,5]. Understanding pain mechanisms allows for a more comprehensive approach to diagnosis and treatment, emphasizing both peripheral and central mechanisms underlying musculoskeletal pain conditions and helping to tailor a multidisciplinary approach.

To date, a variety of treatment options are available to manage pain and reduce disability progression, including pharmacological and non-pharmacological approaches (i.e., physiotherapy, exercise programs and the use of therapeutic modalities) [3].

Contrast Therapy (CT) consists of the repeated application of cryotherapy and thermotherapy, appropriately modulated and timed.

The physiological mechanisms of this therapy involve various systemic responses. Regarding the vascular system, heat induces vasodilation, improving blood flow and promoting tissue healing, while cold causes vasoconstriction, reducing inflammation and swelling, thus aiding recovery [6]. Alternating between heat and cold enhances circulation, accelerating the healing process.

The neurophysiological effects of cold slow nerve conduction through the gate control mechanism, provide short-term pain relief, while heat raises pain thresholds and stimulates the release of endorphins, leading to long-term pain relief and reduced muscle spasms [7,8,9]. Additionally, cold reduces inflammation by lowering cytokine production and inhibiting COX enzyme activity, whereas heat promotes tissue repair by supporting collagen synthesis [10]. This combination of effects may help to resolve inflammation and enhance tissue regeneration.

Furthermore, heat increases tissue elasticity and reduces muscle tension, while cold decreases muscle tone and spasms, improving mobility and flexibility [11]. Beyond its role in sports recovery, performance enhancement, and the treatment of delayed onset muscle soreness (DOMS), CT has been studied in various clinical settings. In acute injuries, cold reduces inflammation and heat supports healing, while in chronic conditions (e.g., osteoarthritis), heat improves circulation and promotes muscle relaxation, whereas cold helps modulate pain and manage ongoing inflammation [6,12,13,14].

The interplay of vasoconstriction, vasodilation, and neural responses is central to this process. Cold exposure causes blood vessels to constrict, reducing blood flow, minimizing heat loss, and preventing tissue damage. This activates the sympathetic nervous system, releasing norepinephrine. Vasoconstriction also limits inflammation and swelling [15,16]. In contrast, heat dilates blood vessels, boosting circulation, oxygen delivery, healing, and reducing muscle spasms [17]. Both stimuli trigger neural responses cold numbs by slowing nerve conduction, while heat enhances blood flow and promotes endorphin release for pain relief. Alternating hot and cold creates a ‘pumping’ effect, improving circulation and controlling pain and inflammation [6].

CT can be administered through various methods, including immersion in contrast baths, the application of ice packs and heating devices, and the use of other external sources [18]. New technology has replaced traditional contrast bath therapy with compact, versatile devices that alternate heat and cold in a single, easy-to-use unit [6]. These new devices are controlled and programmed to provide heat and cold using Peltier elements [19]. Such a device can provide rapid and quantitative stimulation of localized heating and cooling in 0.1° increments, allowing standardization and optimal management of the temperature protocol.

From a safety perspective, these devices help to prevent complications such as heat and cold burns and provide rapid functional recovery or pain relief [13]. Another advantage of these new CT modalities is their efficiency in terms of mobility and temperature maintenance which allows a quantitative assessment of the effects of temperature changes [20].

CT therapeutic applications include some of the most common musculoskeletal degenerative conditions and injuries, such as exercise-related muscular disorders (EAMD), post-surgery pain management, sports injuries and chronic musculoskeletal pain [11].

To date, several research groups have studied the effects of CT to clarify its potential beneficial effects, such as improving overall functionality, reducing fatigue and pain [18,21,22].

The literature on the use of CT is constantly evolving. In recent years, it has attracted increasing attention due to its potential therapeutic benefits, including pain relief, improved mobility, and its possible influence on recovery time. However, the strength of this evidence is mixed, with some studies reporting mild to moderate improvements in pain and function, while others show no significant benefits over standard treatments (e.g., continuous cold or heat therapy alone) [13,21,23]. Several studies on athletes and non-athletes with acute injuries (e.g., sprains, muscle strains) suggest that CT may reduce recovery time compared to using only cold or heat therapy [24,25]. For osteoarthritis, CT can provide short-term relief from pain and stiffness and help to improve mobility [12,26]. However, even though CT is widely used in this context and is considered a first-line intervention in pain management, there is less evidence to suggest long-term improvements in joint health or structure [27,28].

While contrast therapy is widely used, its efficacy remains controversial due to variability in outcome measures across studies. There is a significant gap in knowledge regarding the standardization of CT application, as there are currently no well-defined guidelines for optimal temperature ranges, duration, or frequency of treatment. In addition, much of the existing literature lacks specificity regarding conditions for which CT may be most effective, making it difficult to draw clear conclusions about its clinical utility.

The aim of this scoping review is to summarize the available literature on CT in the management of musculoskeletal painful conditions. Specifically, this review attempts to (1) identify and synthesize the physiological mechanisms underlying the effects of CT, (2) explore the clinical use of CT in different musculoskeletal conditions, (3) analyze the methodological quality and heterogeneity of existing studies, and (4) highlight research gaps to guide future investigations and clinical guidelines. Considering the general exploratory purpose of scoping reviews, the authors aim to provide a synthesis of the current evidence through a structured literature review and to identify applicability in musculoskeletal rehabilitation.

## 2. Materials and Methods

### 2.1. Protocol

This review of the literature on the use of CT in musculoskeletal painful conditions management was structured according to the Preferred Reporting Items for Systematic reviews and Meta-Analyses extension for Scoping Reviews (PRISMA-ScR) [29]. The criteria for the PICOS components are shown in Table 1.

### 2.2. Search Processing

Authors searched for English articles published from 2004 up to December 2024 on the electronic databases PubMed, Scopus and Cochrane Library using the following keywords, combined to maximize the sensitivity of the search strategy: (“contrast therapy”) or (“heat and cold therapy”) or (“contrast bath”) or (“cryo-thermal”) or (“thermal shock”).

### 2.3. Inclusion and Exclusion Criteria and Selection of Sources of Evidence

**Figure 1:**
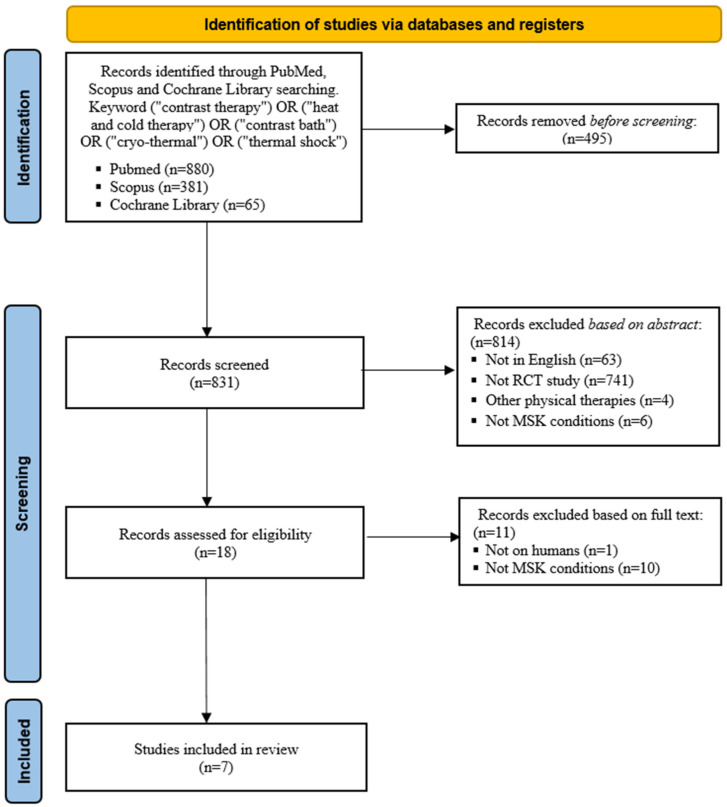
Flowchart summarizing the selection process of papers, with the preferred reporting items for systematic reviews and meta-analyses.

Two independent observers (GL and AA) performed the screening and analysis separately. Firstly, articles were screened by title and abstract, using the following inclusion criteria for selection: (1) randomized controlled trials (RCTs); (2) written in English language; (3) published in indexed journals from 2004 to 2024; and (4) dealing with the use of CT in the management of musculoskeletal degenerative conditions, painful condition and injuries. The exclusion criteria were (1) non-randomized trials; (2) reviews; (3) papers written in other languages than English; and (4) data not dealing with the treatment of musculoskeletal system disease. Secondly, the full texts of the selected articles were screened with further exclusions according to the criteria described above. A PRISMA flowchart of the selection and screening method is provided in Figure 1 (Preferred Reporting Items for Systematic Reviews and Meta-analyses flowchart resuming the paper’s selection process). Any discrepancies were discussed with the senior investigator (SP), who made the final judgment. The results are reported in narrative form, supported by graphs and tables.

### 2.4. Data Extraction

Authors extracted and collected the relevant data into a single database with the agreement of both observers: (1) study design, (2) musculoskeletal pathology, (3) sample size and patients’ features, (4) outcome measures, (5) therapeutic protocol and follow-up, (6) a summary of clinical results, and (7) overall performance (Table 2 and Table 3).

### 2.5. Quality Assessment

Two independent researchers assessed the risk of bias (GL, AA), and disagreements were resolved by a discussion with a third author (SP). The Cochrane Risk of Bias for RCT tools was used for the randomized controlled trials included in the review. The Cochrane Risk of Bias Tool assesses internal validity by systematically evaluating seven key areas. These include randomization sequence, generation allocation, concealment, selective reporting, blinding of participants and personnel, blinding of outcome assessment, incomplete data results, and other biases. The results were then converted to Agency for Healthcare Research and Quality (AHRQ) standards, which finally classify RCTs as ‘good quality’, ‘fair quality’ or ‘poor quality’.

Each study was assessed for potential bias based on the different components specified in the tool.

**Table 2:** Demographic and clinical characteristics of patients: data extracted from studies included.

Publication	Pathology	Score	Patients Features
Fokmare et al., 2023 [13]	Knee osteoarthritis	VAS; WOMAC; two-minute walk test	60 (30 vs. 30) Sex: 23 F 7 M vs. 21 F 9 M Age: 50.06 ± 6.66 vs. 51.43 ± 4.88
Sawada et al., 2022 [6]	Shoulder stiffness	11-point numerical rating scale for muscle fatigue (0 to 10, 0 = not at all, 10 = very much); portable muscle hardness meter; thermocouple	20 M Age (years): 20.3 ± 0.6
Colantuono et al., 2022 [30]	EAMD	VAS; PPT measured by an algometer	10 M Age (years): 21.5 ± 2.7
Weerasekara et al., 2016 [14]	Ankle sprain (grade I and grade II) after the fifth day of injury	VAS; measurement of ankle volume using tank and of ankle ROM using a goniometer	115 (46 vs. 69) Age (years): age limit of 13 to 50 years of both genders, mean (SD) 22.21 (6.93) Sex: M: 86 (73%) F: 31 (27%)
Bilgili et al., 2016 [31]	CRPS Type I in the upper extremities	VAS; LANSS; DN-4; functional capacity using a hand dynamometer and the DHI; measurement of wrist ROM using a goniometer and of hand edema using volumeter	30 (16 vs. 14) Age (years): 49.07 ± 10.26 vs. 45.20 ± 17.65 Sex: M: 14: F: 16
Denegar et al., 2012 [26]	Knee osteoarthritis	KOOS; VAS	34 Age (years): 61.76 ± 14.59 Sex: M: 11 F: 23 4.87 ± 10.67 vs. 54.6 ± 19.91
Denegar et al., 2010 [12]	Knee osteoarthritis	KOOS; VAS	34 Age (years): 62 ± 14 Sex: M: 11 (32%) F: 23 (68%)

CRPS, Complex regional pain syndrome Type I; DN-4, Douleur Neuropathique en 4 Questions; DHI, Duruöz Hand Index; EAMD, exercise-associated muscle damage; F, female; KOOS, Knee Injury and Osteoarthritis Outcome Score; LANSS, Leeds Assessment of Neuropathic Signs and Symptoms scale; M, male; PPT pressure pain threshold; ROM, range of motion; VAS, visual analogical scale; WOMAC, Western Ontario and McMaster Universities Arthritis Index.

## 3. Results

### 3.1. Study Selection

The database search identified a total of 1326 relevant publications. A total of 495 duplicates were excluded from the search before screening. After reading the title and abstract, 814 articles were excluded because they were non-English (n = 63), non-RCTs (n = 741), included other physical therapies (n = 4), and were used to treat other conditions (n = 6). The remaining 18 articles were read in full and 11 were excluded for the following reasons: did not involve human subjects (n = 1), CT was not used for musculoskeletal injuries (n = 10). Finally, seven articles were included in this review.

### 3.2. Study Features

According to the inclusion criteria, all studies were RCTs. A total of 7 studies, published between January 2004 and December 2024, and involving 303 patients with musculoskeletal painful condition treated with CT, were included in this review. From the literature review, musculoskeletal disorders treated with CT in the included studies were shoulder stiffness, exercise-associated muscle damage (EAMD), ankle sprain (grade I and grade II) after the fifth day of injury, Complex regional pain syndrome Type I (CRPS Type I) in the upper extremities and knee osteoarthritis. Baseline and follow-up assessments were based on clinical scores, the most used being the Visual Analogue Scale (VAS) and the Knee Injury and Osteoarthritis Outcome Score (KOOS), as well as measurements of joint ROM using a goniometer and pressure pain threshold (PPT) using an algometer. A detailed description of each study is reported in Table 2 and Table 3.

**Table 3:** Descriptive analysis of the interventions.

Publication	Study Design	Therapeutic Protocol and F-Up	Results	Overall Performance
Fokmare et al., 2023 [13]	RCT (CT for 20 min vs. KPD for 20 min; Otago exercise program was given in both groups for 30 min)	CBT for 20 min/day and Otago exercise for 30 min/day vs. KPD for 20 min/day and Otago exercise for 30 min/day. 3 sessions per week for 2 weeks. Outcome measures assessed at baseline and post-treatment	Group B showed more significant improvement when compared with group A. The enhancement in VAS, ROM, WOMAC and two-minute walk test showed improvement in functional ability	Both groups showed improvement following treatment, but the use of a knee pad device in combination with strengthening and balance retraining is more efficient in reducing pain and enhancing quality of life in patients with grade 1 or 2 knee OA than conventional CBT
Sawada et al., 2022 [6]	RCT with crossover design (Heat and cold stimulation vs. heat stimulation vs. cold stimulation vs. no stimulation)	Alternating heat and cold stimulation vs. heat stimulation vs. cold stimulation vs. no stimulation. Each intervention was administered at least 1 week apart.	After a 30 min typing task, no significant difference in muscle hardness was found for all conditions. However, the muscle hardness in the heat and cold stimulation condition showed a significant decrease	Shoulder stiffness: heat and cold stimulation + heat stimulation = cold stimulation = no stimulation =
Colantuono et al., 2022 [30]	RCT with repeated-measures crossover (within-within) design (CwC therapy pre- and post-exercise vs. no therapy)	2 bouts of eccentric elbow flexor exercise, separated by 1 week; after each bout, participants received either CwC therapy (at 0, 24, and 48 h post exercise) or no therapy	Beneficial effect of CwC over time with significant interaction effects for muscular strength, muscular power, intramuscular glycogen, creatine kinase, muscle thickness, muscle soreness and active elbow flexion. No significant interaction effect for PPT or passive elbow extension.	Glycolysis-dependent athletes may benefit from CwC therapy after training/competition that causes EAMD.
Weerasekara et al., 2016 [14]	RCT (heat therapy vs. contrast therapy)	Heat therapy vs. contrast therapy. F-up for 3 consecutive days (fifth, sixth, and seventh days after the injury)	Heat therapy reduced pain and improved ankle ROM over CT. CT reduced ankle volume at the end of all 3 days; both modalities increased ankle swelling	heat and CT + (ankle pain) CT + (ankle volume) heat and CT = (ankle ROM)
Bilgili et al., 2016 [31]	Double-blinded RCT (conventional TENS therapy + contrast bath vs. sham TENS therapy + contrast bath)	TENS + contrast bath + whirlpool bath + exercise program vs. Sham TENS + contrast bath + whirlpool bath + exercise program. Therapy was scheduled for 15 sessions. All measurements were performed at baseline and after therapy.	Significant improvements in spontaneous and neuropathic pain scores, edema, ROM, and functional capacity in both groups (greater in group 1 regarding pain intensity, neuropathic pain, oedema, and in the 2nd–3rd finger ROM measurements)	The addition of TENS to the physical therapy program was seen to make a significant contribution to clinical recovery in CRPS Type 1 +
Denegar et al., 2012 [26]	RCT with crossover design (using a within-subject, randomized order design, patients received each treatment in 1-week blocks)	Five treatment protocols including cold, warm, contrast with the water-circulating, wrap-around garment system, superficial heat with an electric heating pad or control (rest) twice daily (morning and evening) for 5 days. KOOS and VAS were completed at baseline and twice each week	Women reported clinically meaningful improvement in pain and symptoms on the KOOS with the use of heat, cold, and a heating pad. Men and women reported improved quality of life with intervention.	Pain and symptoms associated with knee osteoarthritis (OA) Pain + (>in women)
Denegar et al., 2010 [12]	RCT with crossover design (cold vs. warm vs. contrast with the water circulating system vs. superficial heat with an electric heating pad vs. control)	Twice-a-day application of one of the treatments for 5 consecutive days, followed by 2 days of non-treatment. F-up: 1 week. KOOS and VAS were completed at baseline and then twice on the 5th and 7th day of each treatment week	Treatment with the device set to warm was preferred by 48% of subjects. Near equal preferences were observed for cold (24%) and contrast (24%). Pain reduction and improvements in KOOS reported for each treatment but responses were greater with preferred treatments.	Pain and symptoms associated with knee OA Pain +

CRPS, Complex regional pain syndrome Type I; CBT, Contrast bath therapy; CT, contrast therapy; CwC, contrast with compression; EAMD, exercise-associated muscle damage; F, female; KOOS, Knee Injury and Osteoarthritis Outcome Score; KPD, knee pad device; M, male; OA, osteoarthritis; PPT, pressure pain threshold; RCT, randomized controlled trial; ROM, range of motion; TENS, transcutaneous electrical nerve stimulation; VAS, visual analogical scale; WOMAC, Western Ontario and McMaster Universities Arthritis Index; +, better results in the experimental group compared to the control group; =, no differences between experimental and control groups.

### 3.3. CT Applications Modality and Control Group

Study designs were highly variable, since patients in the control groups received different injections or treatments: knee pad device + exercise program [13]; placebo [30]; heat therapy [14]; sham TENS + contrast bath + whirlpool bath + exercise program [31]; in the remaining three studies, the authors used a within-subject randomized order design, in which patients received each of the treatments in one-week blocks: alternating heat and cold stimulation vs. heat stimulation vs. cold stimulation vs. no stimulation in Sawada’s study [6] or cold, warm, contrast with a water-circulating system, superficial heat with an electric heating pad and control (rest) [12,26]. Treatment protocols varied widely in the number and frequency of scheduled contrast sessions. Follow-up times and outcome measures also varied widely.

### 3.4. Reported Clinical Outcome

Sawada et al. [6] showed that after a 30 min typing task, CT promoted subjective improvements in refreshed feeling, muscle stiffness, and fatigue, but also reduced muscle stiffness in healthy young male subjects. Similar results have been reported by Colantuono et al. [30], highlighting the beneficial effect of CT over time in the recovery of muscle function after damaging exercise, suggesting that glycolysis-dependent athletes may benefit from this therapy following training/competition that causes exercise-induced muscle damage.

On the other hand, Weerasekara et al. [14] found a decreasing trend in all study variables (pain, ankle volume, and ankle ROM) between pre- and post-treatment values, proving that the use of different thermal modalities during the transition from the acute to the chronic phase of injury can be suggested as effective treatment options to reduce pain, improve ROM and manage swelling.

Moreover, Bilgili et al. [31] investigated the effect of contrast bath combined with transcutaneous electrical nerve stimulation (TENS) versus TENS alone in the treatment of patients with CRPS Type I and showed significant improvements in spontaneous and neuropathic pain scores, edema, 2nd–3rd finger ROM measurements and functional capacity, which were greater in the study group. Thus, Bilgili’s study [31] concluded that the addition of TENS to the physical therapy program made a significant contribution to clinical recovery in CRPS type 1.

Conversely, no statistically significant differences between the effects of cold, warm and contrast versus superficial heat were found in Denegar’s 2010 study [12]. Specifically, pain reduction and improvements in KOOS subscale measures were demonstrated for each treatment, but responses were greater for preferred treatments, with most patients preferring treatment with contrast. Thus, Denegar’s 2010 study [12] recommends CT within a treatment session as another option in the management of many different musculoskeletal conditions, including knee OA. Later, in 2012, Denegar’s [26] study looked at differences in the response of men and women with knee osteoarthritis to superficial heat, cold, or contrast therapy. They analyzed data from their previous study [12] to better understand the influence of gender on treatment response based on the KOOS and VAS scales and concluded that women in the KOOS reported clinically meaningful improvements in pain and symptoms with the use of heat, cold and superficial heat, while men and women reported improved quality of life with the intervention [26]. Later, in 2023, Fokmare [13] also examined the effect of traditional contrast bath therapy on pain reduction and quality of life improvement in patients with knee OA, comparing it to the use of a knee pad device combined with strengthening and balance re-education. The authors found that both groups showed improvement following treatment, but the combination of the knee pad device with strengthening and balance re-education was more effective in reducing pain and enhancing the quality of life for patients with grade 1 or 2 knee OA than conventional contrast bath therapy.

The above reported results showed that the heat and cold stimulation and heat stimulation conditions promoted greater improvements in subjective symptoms than the no stimulation condition. Moreover, the heat and cold stimulation condition showed significantly greater improvements in muscle stiffness than the cold condition. In contrast, the cold condition did not promote significantly greater improvements in subjective symptoms as compared to the no stimulus condition.

### 3.5. Quality of the Included Studies

A total of 7 trials, published between 2010 and 2023, were included in this scoping review, which looked at summarize recent studies on its use for musculoskeletal injuries, evaluating its healing potential in comparison to other conservative therapies. Table 1 and Table 2 show the clinical and descriptive data of the included studies. Looking at the quality of the available literature by the AHRQ standard, we found that all the RCTs included in this revision reached a “Poor quality”.

The random sequence generation was specified in six studies [12,13,14,26,30,31], as well as the method of allocation concealment, which was described in five studies [12,13,14,26,31]. Four studies were RCT with crossover design [6,12,26,30], two studies were RCT [13,14] and one study was double blinded RCT [31]. In addition, the risk of attrition bias was low for all the RCTs included. Moreover, in three studies, it was clearly stated how many patients were screened, how many were excluded from randomization and why, and how many were lost to follow-up, specifying the reason [13,14,26]. Flow diagrams showing the patient selection process were reported only in three of the seven included RCTs [13,14,26]. Finally, we found that only two protocol trials were registered in a public registry [6,13] which should be mandatory according to the Consolidated Standards of Reporting Trials (CONSORT) 2010 guidelines. Table 4 shows the domains that were assessed for each study.

### 3.6. Complications

There were no reports of serious complications in any of the trials included in this review.

**Table 4:** Assessment of the included studies by using the Cochrane risk of bias tool for RCT and the AHRQ (Agency for Healthcare Research and Quality).

Publication	Random Sequence Generation	Allocation Concealment	Selective Reporting	Other Bias	Blinding of Participants and Personnel	Blinding of Outcome Assessment	Incomplete Outcome Data	AHRQ Standard
Fokmare et al., 2023 [13]	Low	Low	Unclear	Unclear	Unclear	Low	Low	Poor
Sawada et al., 2022 [6]	Unclear	Unclear	Unclear	Unclear	Unclear	Low	Low	Poor
Colantuono et al., 2022 [30]	Low	Unclear	Unclear	Unclear	Unclear	Unclear	Low	Poor
Weerasekara et al., 2016 [14]	Low	Low	Unclear	Unclear	Unclear	Unclear	Low	Poor
Bilgili et al., 2016 [31]	Low	Low	Unclear	Unclear	Low	Unclear	Low	Poor
Denegar et al., 2012 [26]	Low	Low	Unclear	Unclear	High	High	Low	Poor
Denegar et al., 2010 [12]	Low	Low	Unclear	Unclear	High	High	Low	Poor

“Good quality”: All criteria met (i.e., low for each domain); “Fair quality”: One criterion not met (i.e., high risk of bias for one domain) or two criteria, and the assessment that this was unlikely to have biased the outcome, and there is no known important limitation that could invalidate the results; “Poor quality”: One criterion not met (i.e., high risk of bias for one domain) or two criteria unclear, and the assessment that this was likely to have biased the outcome, and there are important limitations that could invalidate the results; Poor quality: two or more criteria listed as high or unclear risk of bias.

## 4. Discussion

To date, the effectiveness of CT remains controversial due to a lack of clinical trials with consistent methodologies, making the results on effectiveness confusing [32]. Based on the reported trials, CT has been shown to have a good short-term outcome, improving recovery of muscle function, and reducing muscle soreness after a harmful bout of exercise [6,30,31]. In addition, intra-session CT has been shown to reduce pain, improve ROM and reduce swelling in several musculoskeletal degenerative conditions and injuries, including knee and ankle OA. As heat and cold are non-invasive, generally safe, and inexpensive treatments, they can be considered as a possible therapeutic option in the management of several musculoskeletal conditions [14,30].

Specifically for the management of knee OA, Denegar [12,26] demonstrated CT effectiveness in reducing pain and improving quality of life. Later, Fokmare [13] agreed that CT, when combined with exercise therapy, offers a therapeutic alternative to medication for alleviating pain and stiffness in knee OA patients.

Protocol comparison shows significant differences in application method, time, and sequence in each hot/cold cycle, and total treatment time. In addition, it is not clear whether the application methods or temperatures used in the research reflect what really happens in clinical practice [18]. This means that it impossible to generalize the results and translate these data into clinical practice. In fact, three of the six studies reviewed [6,30,31] included only healthy young adults, so the results cannot be generalized to the whole population. In addition, the relationship between pain and muscle stiffness in acutely injured muscles could not be tested.

The distinction between CT and ice baths is another aspect that is not well understood in the trials. CT seems to alternate between two extreme temperatures, whereas ice baths do not include heat as part of the therapy. In addition, the ice bath protocols suggest a longer immersion time in cold water than CT. It is therefore possible that this condition leads to more significant physiological changes than CT, the ’whole-body’ effects, which should be better studied [18,33,34,35].

Other studies pointed to newer handheld devices as an attractive alternative to water contrast due to their portability, ease of use, limited cleaning, and ability to target tissue [30]. Among these studies, Kim’s [22] highlighted the efficacy of CT in terms of mobility and temperature control using infrared devices and cryotherapy, features that would benefit clinical applications. However, it remains to be determined whether it is appropriate to treat pain with hand-held devices in a clinical context [36].

Other authors [3,37] have shown that conservative management of musculoskeletal degenerative conditions and injuries may even benefit from thermal modalities in the form of heat, cold and CT, during the inflammatory phase.

Comparing CT with thermotherapy, it has been shown that the latter is generally expected to increase tissue temperature, local vasodilation, and soft tissue extensibility by reducing muscle spasms, promoting pain relief and healing of damaged tissue [22]. It is very interesting to note that, as in Sawada’s study [6], there was no significant reduction in muscle stiffness after thermotherapy, whereas only CT provided an objective measure of muscle stiffness reduction. The reduction in muscle stiffness during CT was probably due to cooling of the skin rather than heating, with the combined intensity of the hot and cold stimuli having a significant effect on soft tissue stiffness [6].

This suggests that cold therapy’s physiological effects help control inflammation, pain, and edema during rehabilitation for acute muscle injuries.

Given the link between skin cooling and reduced muscle stiffness, further research should explore cooling intensity to refine CT protocols and clarify the mechanisms behind muscle stiffness.

In addition, the RCTs included here showed that CT improves general well-being, but the mechanisms are still unknown and warrant further investigation.

### 4.1. Practical Applications

CT has several potential applications in the management of musculoskeletal conditions, particularly for pain relief, inflammation control and rehabilitation in pathological contexts. In chronic conditions such as osteoarthritis, CT can help reduce joint stiffness and improve mobility by modulating blood flow and reducing muscle tension. The alternating application of heat and cold can improve circulation, promote tissue healing, and potentially slow disease progression by reducing inflammatory responses [12,26].

For acute injuries such as sprains or strains, CT can be used to reduce swelling and pain. Cold therapy helps to reduce acute inflammation and limit tissue damage, while heat application can aid recovery by increasing oxygen delivery and facilitating metabolic processes essential for healing. Similarly, in post-operative rehabilitation, CT can help to reduce post-operative swelling and stiffness and, when combined with physiotherapy, improve functional recovery [14,24,30].

### 4.2. Limits

This manuscript has several limitations. Firstly, the limited number of included trials with different conditions, different clinical outcome measures, non-comparable follow-up periods and lack of homogeneity in the data have led to an unreliable evaluation. In addition, the overall modest quality of the studies prevents the development of clear guidelines for the use of CT. Another limitation is the authors’ exclusive focus on research conducted within the last twenty years. Finally, grey literature was not considered, as only published, peer-reviewed RCTs were included to minimize potential methodological bias.

## 5. Conclusions

This review highlights the lack of clear guidelines on the applicability and usefulness of CT in the management of painful acute or chronic musculoskeletal conditions. Furthermore, there is a lack of standardization in the CT protocols used across trials (e.g., temperature range, duration, and frequency), making it difficult to compare results across trials. In addition, the trials reviewed have methodological inconsistencies and do not address the long-term benefits of CT for acute injuries, raising concerns about their clinical relevance and making it difficult to conclusively recommend CT over other established recovery methods such as R.I.C.E. (Rest, Ice, Compression, Elevation). Ultimately, although this is a scoping review of RCTs, the modest quality of the trials does not allow the authors to draw clear conclusions about the comparative effectiveness of CT compared with other conservative approaches. However, this study shows that the use of CT should not be discouraged. In fact, the combination of CT’s key benefits, such as improved circulation, reduced inflammation, pain relief and faster recovery, could serve as a complementary treatment option in pain management and functional recovery. Further large-scale studies are needed to investigate the optimal ratio and intensity of cooling in CT, with a focus on determining the most effective protocol for reducing muscle stiffness and timing of intervention. Research should also aim to determine the ideal duration of treatment and identify the patient populations most likely to benefit from this therapeutic approach, particularly in the context of both acute and chronic musculoskeletal conditions.

## Figures and Tables

**Table 1:** PICOS Criteria.

Criteria	Application
Population	Patients with musculoskeletal condition
Intervention	Contrast Therapy
Comparisons	Before and after intervention
Outcomes	Technique-related improvements
Study design	RCT

## Data Availability

The data presented in this study are available within this article.
